# Neurodegeneration progresses despite complete elimination of clinical relapses in a mouse model of multiple sclerosis

**DOI:** 10.1186/2051-5960-1-84

**Published:** 2013-12-23

**Authors:** David W Hampton, Andrea Serio, Gareth Pryce, Sarah Al-Izki, Robin JM Franklin, Gavin Giovannoni, David Baker, Siddharthan Chandran

**Affiliations:** 1 Centre for Clinical Brain Sciences, MS Centre, University of Edinburgh Chancellor’s Building, 49 Little France Crescent, Edinburgh EH16 4SB, UK; 2 Neuroimmunology Unit, Blizard Institute, Barts and London School of Medicine and Dentistry, Queen Mary University of London, 4 Newark Street, London E1 2AT, UK; 3 MRC Cambridge Centre for Stem Cell Biology and Regenerative Medicine and Department of Veterinary Medicine, University of Cambridge, Madingley Road, Cambridge CB3 0ES, UK

**Keywords:** Multiple sclerosis, Experimental autoimmune encephalomyelitis, Neurodegeneration, Remyelination, Gliosis

## Abstract

**Backgound:**

Multiple Sclerosis has two clinical phases reflecting distinct but inter-related pathological processes: focal inflammation drives the relapse-remitting stage and neurodegeneration represents the principal substrate of secondary progression. In contrast to the increasing number of effective anti-inflammatory disease modifying treatments for relapse-remitting disease, the absence of therapies for progressive disease represents a major unmet clinical need. This raises the unanswered question of whether elimination of clinical relapses will prevent subsequent progression and if so how early in the disease course should treatment be initiated. Experimental autoimmune encephalomyelitis in the Biozzi ABH mouse recapitulates the clinical and pathological features of multiple sclerosis including relapse-remitting episodes with inflammatory mediated demyelination and progressive disability with neurodegeneration. To address the relationship between inflammation and neurodegeneration we used an auto-immune tolerance strategy to eliminate clinical relapses in EAE in a manner analogous to the clinical effect of disease modifying treatments.

**Results:**

By arresting clinical relapses in EAE at two distinct stages, early and late disease, we demonstrate that halting immune driven demyelination even after the first major clinical event is insufficient to prevent long-term neurodegeneration and associated gliosis. Nonetheless, early intervention is partially neuroprotective, whereas later interventions are not. Furthermore early tolerisation is also associated with increased remyelination.

**Conclusions:**

These findings are consistent with both a partial uncoupling of inflammation and neurodegeneration and that the regenerative response of remyelination is negatively correlated with inflammation. These findings strongly support the need for early combinatorial treatment of immunomodulatory therapies and neuroprotective treatments to prevent long-term neurodegeneration in multiple sclerosis.

## Background

Multiple sclerosis (MS) is the most common cause of acquired neurological disability in young adults [1]. In the majority of patients multiple sclerosis has two clinical phases reflecting distinct but inter-related pathological processes: focal inflammation drives activity during the relapse-remitting (RR) stage and neuroaxonal degeneration represents the principal substrate of secondary progression (SP), a stage that has few if any relapses. Despite important and continuing advances in treatment of early RR disease with disease modifying treatments (DMTs) that target the inflammatory response, the absence of therapies for progressive disease represents a major unmet clinical need for patients.

The inability of immunosuppressive therapies to influence progressive disease despite suppressing radiological and clinical measures of inflammation highlights a major unresolved question in disease evolution as to the precise relationship between inflammation and neurodegeneration [2]. Indirect observations such as neuronal injury in normal appearing white matter and epidemiological findings that time to disease progression is age dependent, but independent of number of relapses, suggests that neurodegeneration is at least in part independent of immune-driven inflammation [3,4]. An equally plausible case can be made for interdependency whereby inflammation initiates a cascade of events that is necessary for subsequent neurodegeneration [5-7]. In addition, recognition from experimental models that inflammation can be beneficial for remyelination, that can be neuroprotective, adds a further impetus to a better understanding of the relationship between inflammation and neurodegeneration [8-10].

Chronic relapsing experimental autoimmune encephalomyelitis (crEAE) induced in the Biozzi ABH mouse using spinal cord homogenate recapitulates many of the clinical and pathological features of MS including RR episodes and secondary progression, which is associated with accumulating disability along with inflammatory mediated demyelination, neurodegeneration and remyelination [11-16]. Furthermore the ability to eliminate clinical relapses in EAE, using auto-immune tolerance (analogous to the clinical effect of newer disease modifying treatments for multiple sclerosis) achieved with transient antibody mediated T cell depletion and intravenous myelin antigen administration allows evaluation of the contribution of inflammation to neurodegeneration and regeneration to be studied [17,18]. The emergence of increasingly powerful and effective anti-inflammatory DMTs used in RR disease raises the questions of whether these treatments will prevent progression, does the timing of intervention matter and what is the pathological correlate of any putative effect? Definitive evaluation requires long-term human studies with follow up over many years in different temporal cohorts and is currently unrealistic.

The effectiveness of tolerisation to eliminate further clinical relapses in EAE in Biozzi ABH mice has previously been shown [17-19]. We sought to extend the capability of the Biozzi-EAE experimental system to pathologically dissect the consequences of tolerisation with respect to timing of intervention - using tolerisation at two clinical stages chosen to reflect early and late RR disease - and the systematic quantification of axonal and neuronal loss, as well as myelination status.

We show that although early tolerisation is partially neuroprotective, unlike later intervention, there is an on-going neuronal and axonal degeneration even in the absence of any further clinical relapses. Furthermore there is an association between early tolerisation and increased remyelination. These findings are consistent with both a partial uncoupling of inflammation and CNS neurodegeneration and that the regenerative response of remyelination is negatively correlated with this inflammation. These findings strongly support the need not only for early treatment of patients with MS (that requires further advances in identifying symptoms) but that early combinatorial treatment of immunomodulatory therapies alongside neuroprotective treatments might be key to prevent long-term neurodegeneration in MS.

## Methods

### Animals and surgery

All procedures were performed in compliance with national and institutional guidelines (UK Animals (Scientific Procedures) Act 1986 and the University of London Animal Care Committees). These studies were undertaken to conform with the ARRIVE guidelines as described previously [20].

### Induction of EAE and tolerisation

Female 10–12 week specific pathogen free Biozzi ABH mice, stock bred at Queen Mary University of London, were group housed (n = 8-10) in environmentally-enriched cages in humidity and temperature controlled rooms and were provided access to food and water *ad libitum*. Mice were inoculated as previously described [12,20]. Briefly, mice were injected subcutaneously with 1 mg spinal cord homogenate emulsified in Freunds adjuvant supplemented with 60 mg *Mycobacterium tuberculosis* H37Ra and *M. butyricum* in both hind flanks at day 0 and day 7. Mice were monitored and scored daily as follows: 0 = normal, 1 = limp tail, 2 = impaired righting reflex, 3 = hind-limb paresis; 4 = complete hind-limb paralysis and 5 = moribund/death. Signs of reduced severity were scored at 0.5 less than the indicated grade [12]. Immune tolerance to inhibit further relapsing autoimmunity was induced by intraperitoneal injection of 250 μg YTS191, CD4 depleting monoclonal antibody followed one week later by the intravenous injection of 2 × 10^7^ splenocytes chemically coupled to spinal cord homogenate (Ag-Splenocytes) as described previously [12,17]. All experiments were performed over 3 separate cohorts of mice, each cohort being used to generate different groups or part of the groups (including, normal, day 29, 58 or 105 EAE progression or early and late tolerised).

### Immunohistochemistry

**Figure 1:**
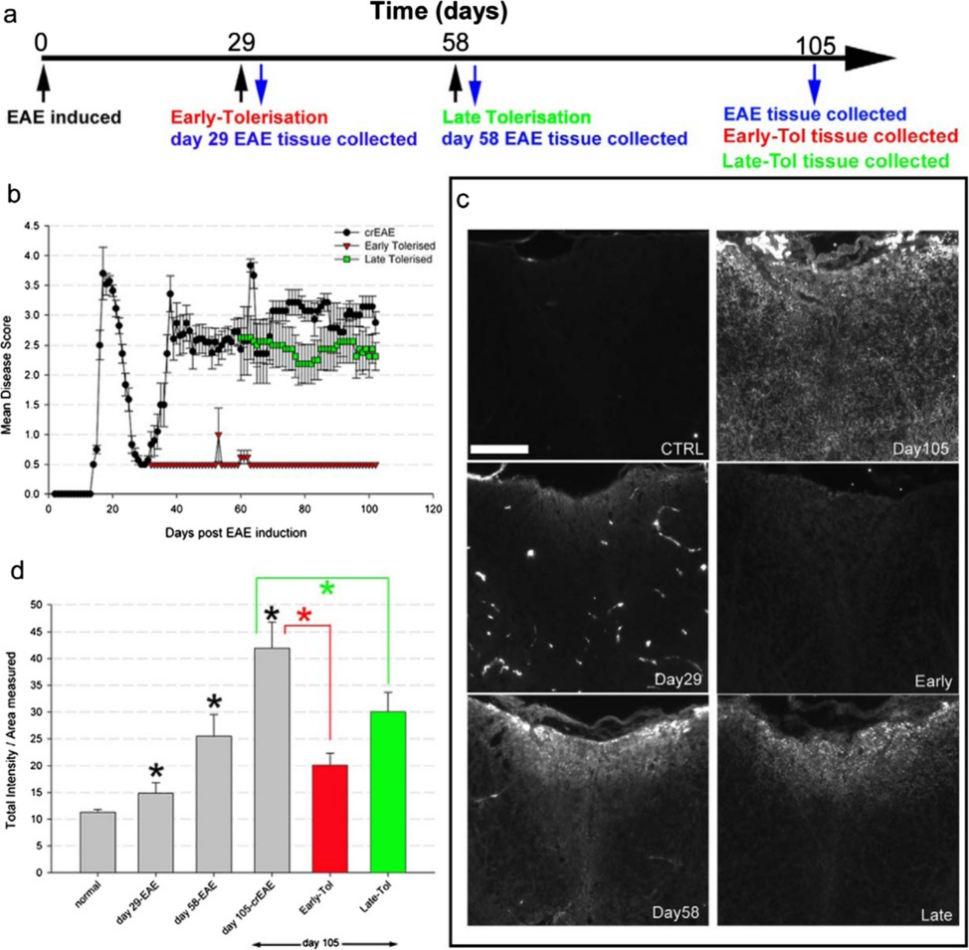
**Timecourse of disease progression and effectiveness of tolerisation on clinical relapses, disease progression and IgG immunoreactivity.** Timeline illustrating experimental groupings following EAE induction (29, 58 and 105 days) and timepoints when tolerisation was instigated and collection of tissue occurred **(a)**. Behavioural data **(b)** showing disease progression of EAE in Biozzi ABH mice (black line) compared to early (red, tolerised day 29) and late (green, tolerised day 58) tolerised mice, where 0 is normal upto 5, moribund. Representative IgG images from the cervical spinal **(c)** cord showing normal (N = 3) compared to EAE disease progression at day 29 (N = 5), 58 (N = 5), 105 (N = 4), early (N = 4) and late tolerisation (N = 6). Quantification of IgG immunoreactivity **(d)** showing there are clear, significant increases in intensity (over the first 200 μm from the surface of the dorsal funiculus down) At day 29, 58 and 105 (black asterisk, d). Following early and late tolerisation there is a significant reduction compared to day 105-crEAE (red and green lines/asterisk respectively d, asterisk = P < 0.01, one-way ANOVA). Scale bar = 200 μm for all images.

Tissue was collected at 3 timepoints as follows - from control EAE, non-tolerised animals at the two time points, day 29 and 58 post EAE induction, as well as at the chronic timepoint of 105 days. Tissue from mice tolerised early (day 29) and late (day 58) was collected at 105 days, as well as tissue from age-matched naive Biozzi ABH mice (i.e. 6 month old mice) – See Figure 1a. All animals were terminally anaesthetised (a lethal dose of sodium pentobarbitone, Euthatal, at 0.3 ml/100 g bodyweight i.p.) and rapidly perfused with cold phosphate buffer saline (PBS) prewash followed by cold 4% paraformaldehyde in PBS. The spinal cord was dissected as individual segments and postfixed in either 4% paraformaldehyde overnight before being cryoprotected in 25% sucrose or postfixed in 4% gluteraldehyde. Cryoprotected spinal cords were sectioned para-sagitally (16 μm) with a cryostat, thaw-mounted onto superfrost-plus glass slides (VWR international, UK), and stored at -80°C. Sections for immunofluoresence were processed as previously described [21,22]. Briefly, slides were defrosted and air-dried for several hours before being washed in PBS and then blocked for one hour using 3% normal goat serum (NGS) or normal horse serum (NHS) depending on secondary antibodies to be used, in a 0.2% Triton-X100 detergent solution of phosphate buffer (TX-PBS). Primary antibodies were applied overnight in TX-PBS containing 1% normal serum, appropriate for the secondary antibody. Primary antibodies used were polyclonal goat-ChAT (1:200, Chemicon UK), polyclonal rabbit-calcitonin gene-related peptide (CGRP) (Sigma, Poole UK 1:4000), monoclonal glial fibrillary acidic protein (GFAP) clone GA5-Cy3 (1:500, Sigma, Poole, UK), polyclonal chicken anti-MAP2 (AbCam, Cambridge UK, 1:1000), monoclonal mouse-NeuN-biotinylated (1:400, Millipore Bioscience Research Reagents UK) and goat polyclonal-IBA1 (1:150, AbCam, UK). After several washes in PBS, secondary antibodies were added for 2 hours in TX-PBS containing 1% NGS or NDS and bis-benzamide (Sigma, 1:4000). Secondary antibodies used were Alexa 488, 555 and/or 647 (Molecular Probes/Invitrogen, UK, 1:1000). Slides were washed in PBS followed by a final wash in Tris buffered non-saline (TNS) before being mounted using fluorosave reagent (Calbiochem, Nottingham, UK).

### Preparation of semi-thin resin sections

Spinal cord sections for resin embedding were taken from the PFA fixed animals and immediately post-fixed using 4% gluteraldehyde in PBS, before being transferred to PBS prior to embedding. Processing was done as previously described [22]. Briefly sections were washed again in PBS, before being placed in 2% osmium tetraoxide (TAAB Labs, Aldermaston, UK) overnight at 4°C. The following day the tissue was dehydrated in a graded series of ethanol washes, followed by propylene oxide and then with propylene oxide/resin mix for 3 hours. Following two immersions in 100% resin (minimum of 6 hours each), sections were embedded individually in beam capsules and hardened at 60°C over 24 hours.

Semithin sections were cut using 6 mm glass knives on a Reichert-Jung Ultracut microtome. These were placed on Polysine slides (VWR international, UK) in a droplet of distilled water and flattened by placing and manually rotating the slide on a heat plate. Once sections were dry, the slide was flooded with toluidine blue (5% in a Borax solution) and heated again until vapours evolved. The stain was removed in a stream of hot water and sections dried on a hot plate. Finally slides were cleared in xylene, before being mounted using DPX solution.

### Preparation of fresh-frozen sections

Tissue was collected at 3 timepoints as follows - from control EAE, non-tolerised animals at day 29 post EAE induction, as well as at the chronic timepoint of 105 days. Tissue from mice tolerised early (day 29) and late (day 58) was collected at 105 days, as well as tissue from age-matched naive Biozzi ABH mice (i.e. 6 month old mice) – See Figure 1a. All animals were euthanized using increasing levels of CO_2_ and rapidly perfused with cold phosphate buffer saline (PBS) prewash and the spinal cord was removed before being snap frozen. Spinal cords were sectioned para-sagitally (16 μm) with a cryostat, thaw-mounted onto superfrost-plus glass slides (VWR international, UK), and stored at -80°C. Sections for CD4 immunofluoresence were defrosted and air-dried overnight before being fixed in ice-cold acetone for 5 minutes and then air-dried for a further hour. Block was applied (5% NGS in PBS) for one hour before monoclonal anti-CD4-directly conjugated to Alexa-488 (1:100, eBioscience, UK) was added with 3% NGS in PBS for 2 hours. After several washes in PBS followed by a final wash in Tris buffered non-saline (TNS), slides were mounted using fluorosave reagent (Calbiochem, Nottingham, UK).

### Analysis

Images were captured using a Zeiss Axiovision microscope and axiovision 4.8 software via a digital camera or a Zeiss LSMZ10 Confocal microscope using Zen 2009 software. SigmaScan Pro 5.0 (SPSS, Chicago, IL) was used for subsequent quantitative measurements. Immunopositive cell density measurements were made on thresholded overlays of transverse spinal cord segment images (in which all pixels overlying immunopositive cells had a greyscale value of 68, and all other pixels had a value of 0), such that average intensity could be converted to density measurements by dividing the output by 68 leading to a scale of 0 (minimum) to 1 (maximum possible reading) being generated. This analysis used an automated thresholding procedure [13,22,23]. GFAP and IBA1 densitometric measurements were made on the resulting combined thresholded overlays as a function of distance from the dorsal surface through the dorsal funiculus. This method of quantification measures the density of immunopositive objects independent of their individual intensities. Following this the data was averaged and presented over the set depth of 100 μm (GFAP) or 300 μm (IBA1) from the surface of the dorsal funiculus.

CGRP analysis was performed through the dorsal horn, on thresholded images as described above and identically to previous methodology [13] except instead of presenting analysis as a function of intensity over depth – data was averaged over 50 μm. NeuN counts were performed in the Lateral Spinal Nucleus (LSN) as defined by overlaying CGRP-MAP2 and NeuN images, such that the LSN is MAP2 positive, but no CGRP positive fibres are present within the LSN [24] and only NeuN positive nuclei that fell within this MAP2 positive area were counted in a total of six areas per animal.

Secondary mouse IgG (Alexa 488) alone images were all generated and handled identically to eliminate personal bias and errors. Once images were captured the raw tiff files were opened in SigmaScan pro 5.0 and the total pixel intensity under a defined area was calculated. A minimum of 3 to a maximum of 6 sections were analysed per animal and 3 measurements were made on each image such that the whole dorsal funiculus, the gracillus or cuneatus alone were measured and quantified.

Semithin sections (n = 5, a minimum of 3 sections per animal) were analysed by counting toluidine blue stained normal myelin, preserved demyelinated axons or thinly myelinated axons (denoting potential remyelination) that dissected a counting line (with a total of 5 counts per section taken) at specified depths through the dorsal funiculus. All counts were performed blinded and data plotted as a percentage of total axons remaining in the cervical spinal cord segment of crEAE mice compared to normal, healthy controls. In addition total area of spinal cord segments was also quantified (n = 4 or 5, a minimum of 3 sections per animal) at all timepoints to examine any potential oedema within the spinal cord tissue.

Graphs were generated using graphing software (Sigma Plot 11) and only statistically significant results are discussed. Statistical tests were performed using SigmaStat and significance only assumed in ANOVA tests using the more stringent Tukey post-hoc Test and a threshold of p = <0.01.

## Results

To fully characterise and control our experiment it was necessary to take multiple animals at key timepoints in disease and treatment (Figure 1a) as follows:

1. Normal control mice

2. EAE disease – day 29, 58 and 105

3. Early-tolerised mice (tolerised at day 29) tissue collected at 105 days

4. Late-tolerised mice (tolerised at day 58) tissue collected at 105 days

During crEAE in Biozzi ABH mice, lesions occur along virtually the entire neuroaxis during active paralytic disease and occurs consistently in the cervical spinal cord regions examined (4,18). However, the amount of immune infiltration is dramatically lost as animals remit (4,12), such that spinal cord can appears relatively normal (4), but residual lesions accumulate with time (4,12). Analysis was undertaken in areas where lesions would have been present, as clearly shown in our previous detailed characterisation studies (18,22).

### Immunological tolerance prevents further relapses in chronic-relapsing EAE

In order to explore the pathological consequences of differential timing of immunosuppresion, immunological tolerance was induced at early (day 29) and late (day 58) time points. These stages of EAE, chosen to mimic common clinical scenarios, represent the recovery stage after the first attack (day 29) and early progressive disease phase with super-imposed relapses (day 58; Figure 1a). Early and late immunological tolerance treatment prevented further relapses, characterised by discrete weight loss and a noticeable increase in neurological score [20]. This halted evolution of clinical deficits, as assessed using the clinical score, from time of treatment initiation, consistent with previous findings, Figure 1b [17]. Any variations in daily clinical score were minor and reflected subtle scoring differences of the subjective scoring system (Figure 1b). Although early treatment results in negligible, long-term residual clinical deficit (Figure 1b, red line), the late treatment group remained significantly disabled with persistent limb paresis (Figure 1b, green line) with no evidence of reversal of disability, again in agreement with previous results [17,19]. Having established a reproducible model in which inflammatory activity manifesting clinically as relapses was silenced in agreement with previous work [17,19] we next undertook detailed quantitative pathological analysis in the cervical spinal cord, an area previously demonstrated as consistently and severely effected during the disease course [11,13,25].

### Timing of immunological tolerance influences immune-reactivity and gliosis

IgG immuno-intensity (Figure 1c-d), used as a surrogate of general immune-reactivity noting its association with microglia [26,27] and blood brain permeability [21,28,29], was examined to further confirm the effectiveness of auto-immune tolerance to halt immune reactivity. The temporal increase in IgG immuno-intensity observed in EAE evolution was halted by both early (red bar, Figure 1d, no significant difference to day 29) and late (green bar, Figure 1d, no significant difference to day 58) tolerisation.

**Figure 2:**
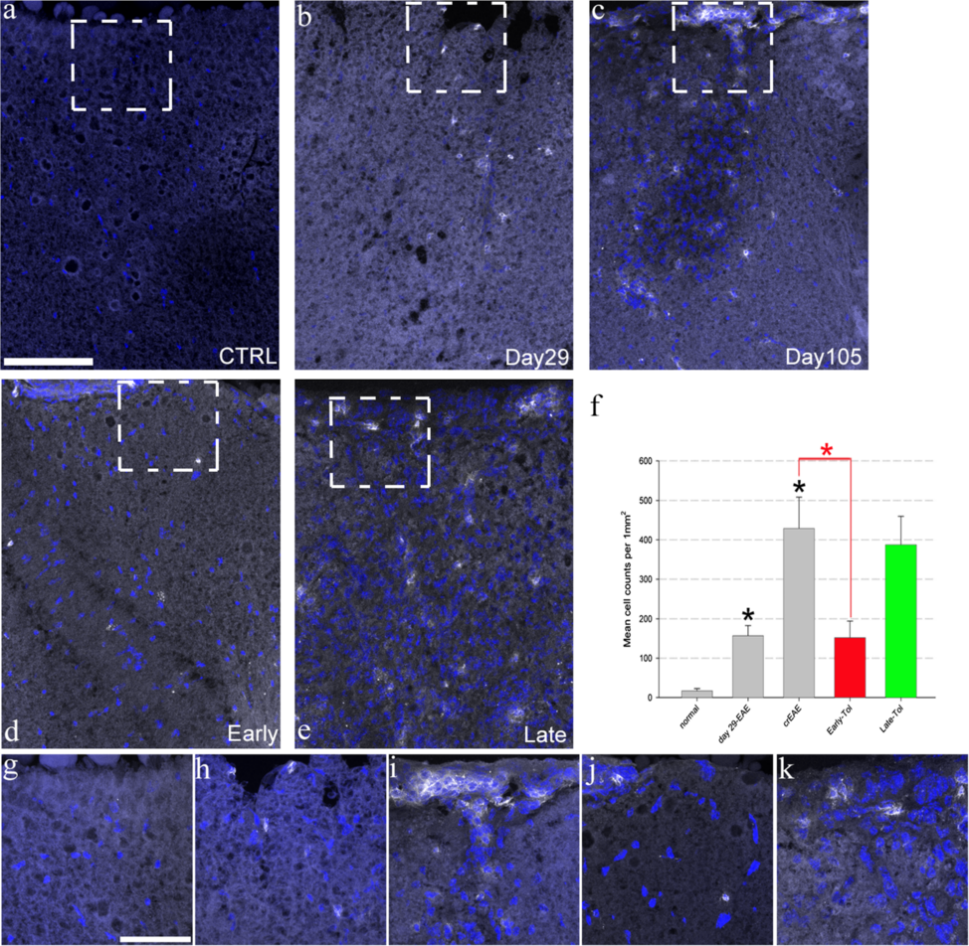
**CD4 positive T-cells increase with EAE progression and early tolerisation significantly reduces them.** Representative maximum projected confocal images of CD4 positive cells (shown in white) and cell nuclei (blue) in the dorsal funiculus, with zoomed in areas denoted by the white dotted-line boxes from normal (**a** and **g** N = 3), day 29 (**b** and **h** N = 4), day 105-crEAE (**c** and **i** N = 3) and following early (**d** and **j** N = 4) and late (**e** and **k** N = 5) tolerisation, Scale bars = 100 μm for images a-e and 50 μm for images **g**-**k**. These images and the quantification **(f)** show a significant, progressive increase in CD4 positive cells penetrating the CNS (black asterisk, P < 0.01, one-way ANOVA), that early tolerisation (red bar, P < 0.01, one-way ANOVA) significantly reduces compared to day 105-crEAE back to a similar number as at day-29. Late tolerisation (green bar, **f**) had no significant effect compared to day 105-crEAE.

CD4 immunopositive reactive T-cells were also quantified (Figure 2) to verify previous publications detailing the effectiveness of this tolerisation technique [17,20]. As clearly shown there is a progression of CD4 positive reactive T–cells at day 29 compared to normal-control naïve mice (a 9.4 ± 1.6 fold increase Figure 2a, b and f) that progresses to a 25.7 ± 4.8 fold increase by day 105 (Figure 2a, c and f). Following early-tolerisation (mice tolerised at day 29 and analysed at day 105), there was a significant reduction in CD4 positive cells when compared to day 105 non-tolerised EAE mice, with the number of positive cells being almost identical to day 29 (9.1 ± 2.6 fold increase compared to normal control mice; Figure 2a, b, d and f). Late tolerisation had no impact on the number of increased CD4 positive cells compared to day 105 EAE tissue (23.3 ± 4.3 fold increase compared to normal control issue for late whilst it is a 25.7 ± 4.8 fold increase for EAE at day 105; Figure 2).

**Figure 3:**
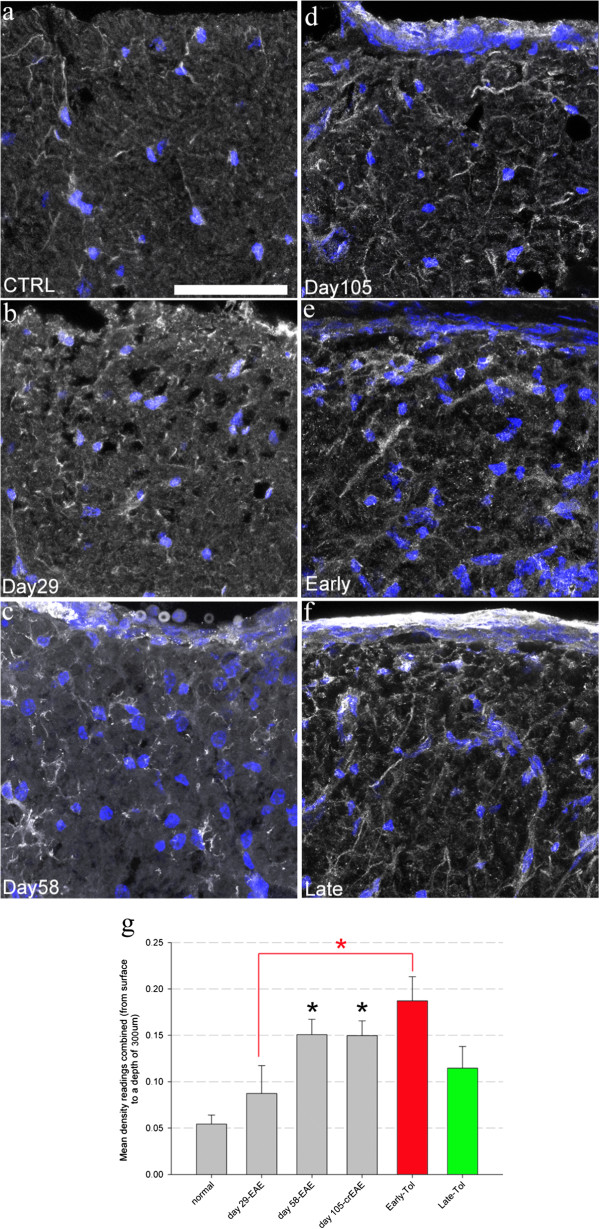
**IBA1 positive, chronically activated microglia increase with EAE progression however both early and late tolerisation have no effect on this increase.** Representative maximum projected confocal images of IBA1 (white) positive microglial cells and bis-benzamide (blue) in the dorsal funiculus in normal (**a**, N = 4), day 29 (**b**, N = 5), day 58 (**c**, N = 5), day 105-crEAE (**d**, N = 7) and following early (**e**, N = 5) and late (**f**, N = 7) tolerisation. Quantification **(g)** shows a progressive increase in IBA1 positive cells in the dorsal funiculus that is significantly elevated at day 58 and 105-crEAE (black asterisk, P < 0.01, one-way ANOVA). Neither early (red bar) nor late tolerisation (green bar) had any significant effect on this increase in IBA1 positive microglia. Scale bar = 50 μm for all images.

IBA1 (ionised calcium binding protein 1) is a calcium binding protein produced exclusively by microglia and activated monocytes and is associated with chronic inflammatory processes. IBA1 positive microglia were seen to increase as disease progressed. Therefore in normal control, naïve mice 0.05 ± 0.01% (Figure 3a, g) of the dorsal funiculus measured (300 μm from the surface down) contained IBA1 positive signal. This increased to 0.08 ± 0.3% at day 29 (not significant, Figure 3b, g), 0.15 ± 0.2% at day 58 (Figure 3c, g) and day 105. Following both early and late tolerisation (mice tolerised at day 29 or 58 and analysed at day 105), there was no impact on IBA1 density measurements when compared to day 105 EAE (0.18 ± 0.3 and 0.12 ± 0.3 respectively, Figure 3d, e and g).

**Figure 4:**
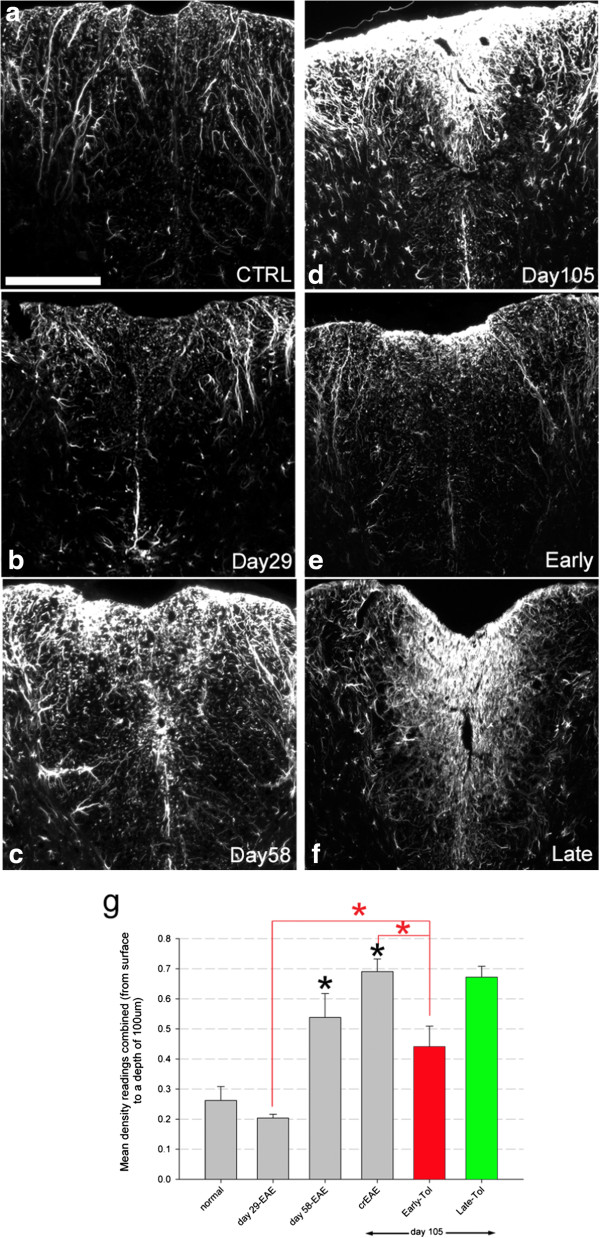
**GFAP reactive gliosis increases with EAE progression.** Gliosis is significantly reduced by early tolerisation but still progresses even in the absence of further clinical relapses. Representative images of GFAP positive reactive astrocytes in the dorsal funiculus in normal (**a**, N = 4) day 29 (**b**, N = 5), day 58 (**c**, N = 5), day 105-crEAE (**d**, N = 7), and following early (**e**, N = 4) and late (**f**, N = 7) tolerisation. Quantification **(g)** shows a progressive increase in GFAP reactivity, (black asterisk) that early tolerisation (red bar and asterisk in g) significantly reduces compared to day 105-crEAE. However it remains significantly elevated compared to day 29, showing increased astrocytosis even in the absence of the primary relapse-remitting driven immune response. Late tolerisation (green bar in **g**) had no significant effect on GFAP reactivity compared to day 58 or 105-crEAE timepoints (asterisk = P <0.01, one-way ANOVA). Scale bar = 200 μm for all images.

In order to examine the influence of early and late tolerisation on gliosis the natural history of EAE was first determined at day 29, 58 and 105 in non-tolerised EAE and compared to age-matched, normal control naïve mice. Disease progression was associated with an increased astrogliosis measured by quantitative GFAP immunohistochemistry. There was a significant increase at day 58 (0.54 ± 0.08) and 105 (0.69 ± 0.07) compared to day 29 (0.20 ± 0.01) and naive mice (0.26 ± 0.05) (Figure 4; p < 0.01), which is in agreement with previous results [13]. Following early-tolerisation (mice tolerised at day 29 and analysed at day 105), there was a significant reduction in GFAP positive cells (0.44 ± 0.07, red bar and line Figure 4g and e; p < 0.01) when compared to day 105 non-tolerised EAE mice (0.69 ± 0.07). However, early tolerised animals (analysed at day 105) still have significantly elevated numbers of GFAP positive astrocytes compared to EAE mice analysed at day 29 (red bar Figure 4g; p < 0.01), despite the complete absence of further clinical relapses (red line, Figure 1b). However GFAP quantification of late tolerised mice showed no difference when compared to non-tolerised EAE mice analysed at day 58 or 105 (green bar, Figure 4g).

### Early immune suppression does not prevent neurodegeneration

**Figure 5:**
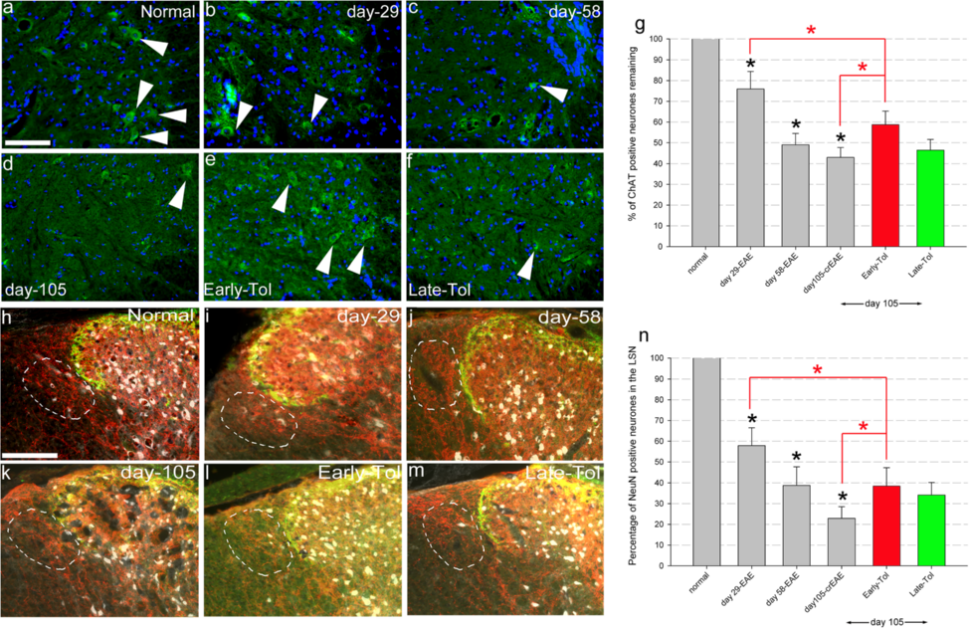
**Neuronal loss following EAE progression is reduced following early tolerisation but still persists despite the absence of further clinical relapses.** ChAT positive motor neurones in the ventral horn in normal, naïve control mice (**a**, N = 3), as EAE progresses at day 29 (**b**, N = 5), day 58 (**c**, N = 5) and day 105-crEAE (**d**, N = 7), following early (**e**, N = 4) and late (**f**, N = 7) tolerisation. Quantification of ChAT **(g)** positive cell counts showing early, progressive neuronal loss. Following early tolerisation neuronal loss was decreased compared to day 105-crEAE (red bar and lines **g**). However this loss was greater then observed at day 29 (ChAT - red bar and lines **g**) showing that neuronal loss is ameliorated however still progresses even in the absence of further clinical relapses. Late tolerisation had no effect on this neuronal loss (**f** and green bar **g**). NeuN positive neurones in the lateral spinal nucleus (LSN), as demarcated by the white dotted lines, again in normal, naïve control mice (**h**, n = 3), as EAE progresses at day 29 (**I**, n = 5), day 58 (**j**, n = 5), day 105-crEAE (**k**, n = 4), following early (**l**, n = 4) and late (**m**, n = 5) tolerisation. Quantification of NeuN **(n)** showing early, progressive neuronal loss. Following early tolerisation neuronal loss was decreased compared to day 105-crEAE (red bar and lines **n**). However this loss was greater then observed at day 29 (red bar and lines **n**) showing that neuronal loss is ameliorated however still progresses even in the absence of further clinical relapses. Late tolerisation had no effect on this neuronal loss (NeuN-m and green bar **n**). Scale bar = 100 μm for all images, significance for data, P < 0.01, one-way ANOVA.

To study the influence of tolerisation on neuroaxonal status quantitative analysis of two classes of spinal cord neurons and axons was undertaken. ChAT positive motor neurone quantification within lamina IX of the ventral horn in the cervical cord showed progressive loss of neurones as EAE developed, at all three time points studied, that is day 29, day 58 and day 105, when these timepoints are compared to normal, control naïve mice. Thus at day 29 there were 75.9 ± 8.5% ChAT^+^ neurons compared to control naïve mice. This progresses to 49.0 ± 5.5% at day 58 and 42.8 ± 4.8% survival at day 105 (Figure 5a-g).

Comparable progressive neuronal loss was also evident in the lateral spinal nucleus (LSN) a longitudinal sensory nucleus found in the dorsolateral white matter involved in homeostatic and noiciceptive signalling [24]. Specifically at day 29–57.9 ± 8.6% NeuN^+^ neurones were present compared to normal, control naive mice with 38.8 ± 8.9% NeuN^+^ at day 58 and 22.9 ± 5.6% by day 105 (Figure 5h-n). These findings are consistent with earlier studies that showed early loss of spinal cord neurones in MS and an alternative form of EAE [30,31].

Having demonstrated progressive neuronal loss in EAE the effect of tolerisation was next studied. Quantification of ChAT^+^ positive neurones in the ventral horn (58.7 ± 6.6% survival in early tolerisation analysed at 105 days compared to 42.8 ± 4.8% survival in non-tolerised EAE mice analysed at the same timepoint of 105 days, Figure 5a-g, red bar and lines; p < 0.01) revealed a beneficial effect of early tolerisation. This beneficial effect was also present in the LSN where an lack of a decrease in NeuN positive neurones was also observed (38.4 ± 8.7% surviving following early tolerisation versus 22.9 ± 5.6% in non-tolerised EAE mice analysed at the same timepoint of 105 days; Figure 5h-n, red bar and lines, p < 0.01).

However, both ChAT and NeuN analysis of early tolerisation revealed a significant ongoing neuronal loss despite the absence of further clinical events following early tolerisation (red line Figure 1b). Therefore counts of both ChAT and NeuN positive neuronal populations show that even in the absence of further clinical relapses in the intervening time (from day 29 to 105) there has been a significant, progressive loss of neurones (Figure 5, red bars and lines; p < 0.01). For ChAT this was a loss of over 15% (75.9 ± 8.5% positive neurones at day 29 compared to 58.7 ± 6.6% surviving in early tolerised mice). A similar loss of around 19% was found for NeuN positive neurones (57.9 ± 8.6% at day 29 dropping to 38.4 ± 8.7% surviving at 105 days following early tolerisation).

### Early immune suppression does not prevent axonal degeneration

**Figure 6:**
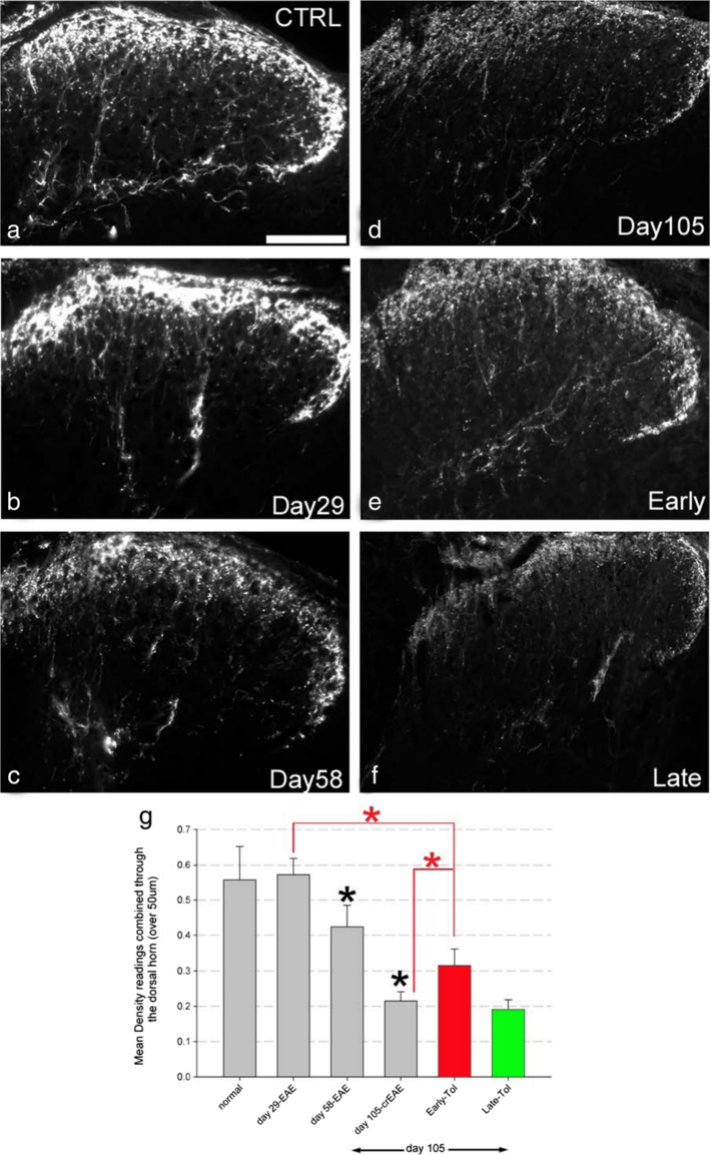
**CGRP positive axonal loss within the dorsal horn develops with EAE, is reduced following early tolerisation but still progresses even in the absence of further clinical relapses.** CGRP positive primary afferents in the dorsal horn in normal, naïve control mice (**a**, N = 4), as EAE progresses at day 29 (**b**, N = 5), day 58 (**c**, N = 5) and day 105-crEAE (**d**, N = 6), as well as following early (**e**, N = 4) and late (**f**, N = 7) tolerisation. Quantification **(g)** shows a progressive loss (black asterisk) over disease course that is reduced following early tolerisation, however still persists when compared to day 29 (**g** - red bar, lines and asterisk). Late tolerisation had no significant impact on progression of CGRP loss (**g**, green bar). Scale bar = 100 μm for all images, significance for all data, P < 0.01, one-way ANOVA.

**Figure 7:**
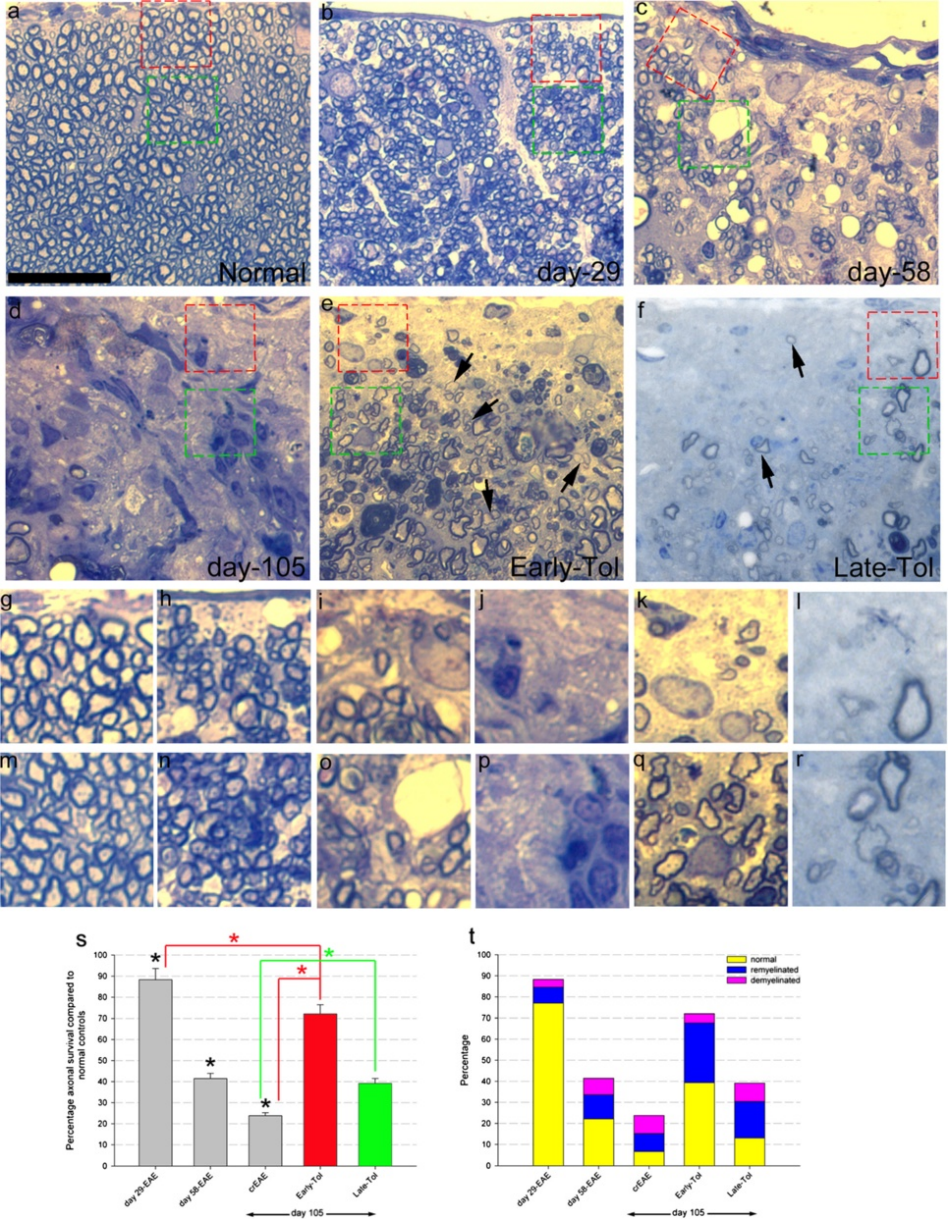
**Axonal loss, demyelination and remyelination progresses following EAE induction.** Following tolerisation axonal loss is reduced however still progresses despite the absence of further clinical relapses. Semithin sections from normal, naïve control mice (**a**, **g** and **m**; N = 5), as EAE progresses at day 29 (**b**, **h** and **n**; N = 5), day 58 (**c**, **i** and **o**; N = 5) and 105 days (**d**, **j** and **p**; N = 8), following early (**e**, **k** and **q**; N = 5) and late (**f**, **l** and **r**; N = 5) tolerisation. Panels **g**-**l** and **m**-**r** are higher magnification images from the red and green boxes respectively in panels **a**-**f**. Quantification shows progression of axonal loss **(s)**, with total axonal survival decreasing as EAE progresses, black asterisks **(s)**. Early tolerisation reverses this trend with significant axonal survival (red bar, **s**) and late tolerisation also has a small but significant effect on axonal survival compared to day 105-crEAE (green bar, **s**). However, following early tolerisation there is still a significant decrease in axonal survival observed when comparing day 29 to early tolerised mice (**s**, red bar and asterisks). The majority of those axons surviving at day 29 were normally myelinated (i.e. unaffected) as shown in panel **t**, yellow segment. Following early tolerisation a significant increase in these remaining normally myelinated axons compared to day 105-crEAE was observed, but a significant decrease compared to day 29 (**t**, yellow segments) showing a progression on axonal degeneration, even in the absence of the primary immune response. Furthermore remyelination (examples shown by the black arrows in **e** and **f**) is significantly increased in early, and to **a** lesser extent late, tolerised mice by the blue segments **(t)**. Scale bars = 25 μm **(a-f)** and 5 μm **(g-r)**, significance for data, P < 0.01, one-way ANOVA.

In order to address whether tolerisation also influenced axonal status we undertook multiple methods of analysis as we did for neuronal status. CGRP quantification within the dorsal horn (to identify primary afferent processes [13,24]) and semithin analysis within another region of the cervical spinal cord, the dorsal funiculus, predominantly comprised of ascending primary sensory afferents [13,24]. Both methods of analysis showed progressive and significant axonal loss as disease progressed, as also observed for neuronal loss. For CGRP positive processes no loss was observed at day 29 EAE mice (0.57 ± 0.05) compared to normal, control naive mice (0.56 ± 0.1). However by day 58 of EAE progression CGRP had significantly decreased to 0.41 ± 0.05 and then progressed further to 0.21 ± 0.03 by day 105 (Figure 6a-f, quantified in g). Counts of semi-thin resin sections of spinal cord, showed that at every time point studied in the non-tolerised EAE mice compared to control naive mice there was progressive loss. Therefore at day 29 a loss of 11.7% ± 5.4, 58.6% ± 2.5 loss at day 58 and 76.2% ± 1.4 loss at day 105 (Figure 7a-f and linked zoomed areas g-l and m-r, quantified in s; p < 0.01).

Further analysis of axonal numbers by both CGRP and semithin methods again revealed a protective effect with early tolerisation. CGRP density measured in early tolerised mice (i.e. mice tolerised at day 29 and analysed at day 105) showed 0.32 ± 0.05 compared to a loss of 0.21 ± 0.03 in day 105 non-tolerised EAE mice (red bar and line Figure 6g). Semithin analysis showed a 28% ± 4.4 loss in early tolerised mice analysed at day 105 compared to 76.2% ± 1.4 loss in day 105 non-tolerised EAE mice (red bar and line Figure 7s). However although both CGRP and semithin analysis showed an axonal protective effect there was still on-going significant progressive axonal loss when comparing early tolerised mice analysed at day 105 to day 29 (Figures 6g and 7s red bar, lines and asterisks), the same effect as was observed for ChAT and NeuN positive neurones.

Total spinal cord area measurements excluded tissue oedema and/or atrophy accounting for the observed changes in axonal counts (Additional file 1: Figure S1). Interestingly late tolerisation had no axonal-protective effect in CGRP analysis in the dorsal horn (Figure 6d, f and green bar in g) but did confer an axonal-protective effect although more modest than early tolerisation at 60.8 ± 2.4 compared to 76.2% ± 1.4 for crEAE (p < 0.01, green bar and line, Figure 7) for semithin analysis in the dorsal funiculus.

Our results therefore show subtle differences between the effect of early tolerisation on different neuronal and axonal areas as well as differences between early and late tolerisation and this in part is because different tracts and/or neuronal areas are impacted at different times during disease progression (Additional file 2: Figure S2). For example CGRP positive axons in the dorsal horn show no loss at day 29, but a loss at 58 days of EAE progression, with progressive axonal loss even following early tolerisation (Additional file 2: Figure S2b).

### Inhibition of relapsing autoimmunity by immune tolerance is associated with increased remyelination

Having established that early tolerisation reduces but does not stop neuroaxonal loss, the influence of tolerisation on myelination status (demyelinated, remyelinated or normally myelinated) of surviving axons was investigated. Quantitative semithin analysis of spinal cord sections revealed a significant reduction in normally myelinated axons in non-tolerised EAE from 77.0 ± 6.9% at day 29 to only 6.7 ± 3.6% by day 105 (Figure 7a, balong with linked magnified panels in g, m and yellow segments in t). Early tolerisation, analysed at day 105, resulted in a significant reduction in this loss of normally myelinated axons (39.4 ± 7.3% compared to 6.7 ± 3.6% of non tolerised EAE mice at day 105, yellow segments in Figure 7t; p < 0.01). However, again this proportion was significantly less than that at day 29 in non-tolerised EAE alone (39.4 ± 7.3% compared to 77.0 ± 6.9%, Figure 7t; p < 0.01). Late tolerisation had no significant effects on the proportion of normally myelinated surviving axons (13.1 ± 4.9%, yellow segments in Figure 7t when compared to either day 58 or 105 non-tolerised crEAE, Figure 7t).

Noting the comparatively small percentage of surviving, demyelinated axons in all study groups (pink bars in Figure 7t) we next determined whether neuroprotection associated with tolerisation was related to remyelination status. Quantification of remyelinated axons, identified morphologically as axons with thin blue encircling layer of myelin compared to normal thick myelin, showed that early tolerisation significantly increased remyelination compared to both day 29 and 105 in non-tolerised EAE (28.4 ± 4.1% versus 7.6 ± 2.9% at day 29 and 8.5 ± 2.9% at day 105; blue segments in Figure 7t; p < 0.01). Late tolerisation also showed a more modest, but nonetheless, significant promotion of remyelination compared to day 58 non-tolerised EAE (17.3 ± 3.7%, versus 11.5 ± 2.0% at day 58; p < 0.01; blue segments in Figure 4t).

Taken together, these findings reveal a progressive neurodegeneration despite suppression of the CD4 driven, myelin targeting immune responses that is also associated with enhanced remyelination and that chronically activated microglia may play a key role in this neurodegenerative process.

## Discussion

This study sought to understand the pathological consequences of tolerisation in an experimental model of chronic multiple sclerosis. We show that elimination of clinical relapses by immunological tolerance, even after the first clinical event, although partially neuroprotective is, of itself, insufficient to prevent ongoing and significant neurodegeneration (1,26,36). Furthermore we show that following tolerisation (both early and late, although to a lesser extent in late tolerisation) there is a significant increase in remyelination. These findings are consistent with an uncoupling, in part at least, of autoimmune-driven inflammation and neurodegeneration in evolving disease. This has clear similarities with progressive multiple sclerosis, which also does not appear to respond to peripheral immunosuppression [1,5,17,19,32]. Whilst immunological tolerance induction inhibits peripheral T cell function and controls relapsing disease and accumulation of T cells within the CNS [17,18], as tolerance induction is dependent on the presence of regenerating T cells, it is not possible to totally exclude a role of T cells in the development of progressive neurodegeneration, especially through a central action involving glial inflammation [7]. Although early tolerisation resulted in minimal clinical deficit it is important to recognise that the clinical behavioural scale used for EAE is ordinal and insensitive to subtle but significant clinical deficits. Consequently although valuable it may not directly translate to patient disability and emphasises the importance of histological quantification of actual neuroaxonal damage.

Our study extends earlier reports that Biozzi ABH EAE faithfully reproduces many key temporal, clinical and pathological features of MS [12,13,19]. Specifically the temporal evolution of early clinical relapses followed by progression, independent of relapses, associated with accumulating demyelination, remyelination, gliosis and neuroaxonal degeneration supports its use to model RR-SPMS. The effectiveness of tolerisation to eliminate further clinical relapses has previously been shown and characterised [17-19] and we further verify the effectiveness of this technique to silence the CD4 positive, T-cell driven EAE in the Biozzi ABH mouse. Notwithstanding our results and these earlier studies of tolerisation [17-19] we now provide additional evidence that abolition of clinical relapses following tolerisation is associated with reduced immune responses measured by reduction in IgG immunoreactivity noting that IgG immunoreactivity is used as a measure of microglia [18,26] and also blood brain barrier disruption/permeability [21,28,29]. Taken together, the ability to reliably eliminate clinical relapses along with pathological surrogates of inflammation using an established immune based tolerisation strategy accurately mirrors current clinical scenarios where second-generation DMTs are able to prevent further clinical and radiological measures of inflammation [32,33]. This allowed the question of whether timing of “immune-treatment” alters clinical and pathological course of EAE to be addressed. Resolving this question in humans is self-evidently difficult in terms of pathological assessment despite advances in MR based imaging and definitive clinical evaluation requires many years of follow up given the temporal evolution of disease progression.

Two major observations are evident following early and late tolerisation. Firstly effective suppression of clinical and immunological measures of inflammation even after the first clinical event, although beneficial and significantly more effective than late tolerisation in respect to neuroaxonal status, does not prevent progressive neuroaxonal loss. We determined this across not only multiple regions of the spinal cord but also using multiple techniques that reveal a consistent pattern across not only ascending tracts and nuclei (the dorsal funiculus and lateral spinal nucleus) but also primary afferent processes within the dorsal horn (CGRP) and ventral motor areas (ChAT positive neurones). This suggests that neurodegeneration can evolve independently of active autoimmunity. Further to this observation we have data that shows that chronically activated CNS microglia (IBA1 positive) may well be involved in this process since neither early nor late tolerisation had any impact on the development of significant increases in IBA1 positive cells (Figure 3) measured after 105 days of progression. Importantly total area measurements of the spinal cord exclude tissue oedema and/or atrophy accounting for the observed changes in axonal counts.

Secondly remyelination appears to be enhanced following tolerisation, with earlier intervention significantly more effective than later tolerisation. This finding is in contrast to other studies that have shown inflammation promoting remyelination [8,10]. This may reflect differences in models; specifically, use of non-myelin driven disease and focal injuries compared to, as in this study, a systemic myelin mediated injury model associated with a progressive neurodegenerative component. Although these experiments were not designed to elucidate the role of remyelination in neuroprotection our findings are consistent with the idea of remyelination being neuroprotective [34-36]. However although we observe that following early tolerisation there is an increase in remyelination there is significant, ongoing axonal and neuronal degeneration as well as an increase in chronically activated microglia. There are other studies that have shown this phenomenon of axonal loss despite extensive remyelination [15]. This is also important since increases in reactive microglia were also noted. So although we cannot definitively say that it is this increase in chronically-activated microglia driving this progressive axonal loss or merely being a consequence of it, there is an increasing amount of literature reporting microglial-induced neurodegeneration in several different models.

A final observation regarding neuronal loss is that comparing early to late tolerisation a small (but still significant) difference was observed in ChAT positive motor neurones (Figure 5g) but not NeuN positive neurones in the LSN (Figure 5n), whilst there are clear behavioural differences between early and late tolerised animals as shown in the EAE clinical scoring. This re-iterates the fact that we analysed multiple areas of the spinal cord and it must be remembered that the EAE scoring system is a motor behavioural task and motor neurones do show a significant difference as well whilst the NeuN positive neurones are in an ascending sensory pathway (the LSN is associated with homeostatic and nociceptive signalling) that is not related to this motor EAE behavioural scoring system. Interestingly the GFAP and semithin analysis were also performed in an ascending sensory area, the dorsal funiculus and also show a significant difference between early and late tolerisation like the ChAT positive motor neurones. However it should be noted that this tract is not exclusively sensory, although it is predominantly comprised of ascending primary afferent axons that are integral to correct motor function. This merely further highlights the complexity of this model showing the need to undertake multiple methods of analysis in an effort to better understand it.

It should also be noted that different axonal tracts and neuronal nuclei are impacted by EAE at different times (Additional file 2: Figure S2). In EAE lymphocytic lesions are concentrated in the white matter however it is clear that there are both white and grey matter influences as we have shown. The main point is that we have taken a constant region (C5) and shown clearly that different nerve tracts are affected at different times during the relapsing course of EAE in Biozzi mice.

Although our studies are undertaken in an adult mouse it would be of interest to examine an older group and extend evaluation beyond 105 days noting that human disease progression is age-dependent [3,4]. Previous studies have indicated that very late tolerisation after two to three relapses does not stop neurological progressive disease [17,19]. These current experiments were initiated in mice aged 2–4 months and maintained for another 3 months which is broadly comparable to a human aged between 20–35 years (based from JaxLabs; webpage address - http://research.jax.org/faculty/harrison/ger1vLifespan1.html). It is therefore likely that our study underestimates long-term equivalent neurodegeneration in a clinically relevant aged human cohort. Furthermore the remyelination that does occur is known to be not as thick as normal myelin [14] and it is also known that age significantly alters not only the remyelination potential but also the injury response to the initial demyelinating episode [37-39]. Therefore by investigating this phenemoenom in aged Biozzi-ABH mice we might be able to elucidate some reasons for the progression of MS in people.

## Conclusions

In summary, these findings highlight that although timing of anti-inflammatory immune based therapies is critical, even intervention after the first clinical event is unlikely to prevent all long-term neurodegeneration. The clinical implications of these findings are that prevention of progression and long-term clinical disability is likely to require both early and combinatorial immune-suppressant and CNS neuroprotective interventions. Furthermore any neuroprotective intervention may well need to address chronically activated microglia that may well be key to this ‘slow-burn’ neurodegeneration that is independent of immune-driven relapses.

## Competing interests

The authors declare that they have no competing interests.

## Authors’ contribution

DWH designed and carried out experiments, analysed data, drafted the manuscript. AS carried out experiments and analysed data. GP designed and carried out experiments, analysed data, drafted the manuscript. SAI carried out experiments, analysed data, drafted the manuscript. RJMF drafted the manuscript. GG drafted the manuscript and provided main funds. DB designed experiments, drafted the manuscript and provided main funds. SC designed experiments and drafted the manuscript. All authors read and approved the final manuscript.

## Authors’ information

David Baker and Siddharthan Chandran: co-senior authors.

## Supplementary Material

Additional file 1: Figure S1 Representative semithin image sections and graph showing that total area measurements of the spinal cord at all time points are not significantly different therefore tissue oedema and/or atrophy cannot account for the observed changes in axonal counts. Representative semithin images from normal (a, N = 3), day 29 (b, N = 4), day 58 (c; N= ) and day 105-crEAE (d, N = 3). Quantification (e) shows no significant differences between any timepoint nor following early or late tolerisation. Scale bar = 1000 μm for all images. Click here for file

Additional file 2: Figure S2 Graphs showing differences between percentage loss of axons and neurones as disease progresses and following tolerisation. Graphs showing varied axonal (a and b) and neuronal (c and d) loss using quantified semithin axonal counts in the dorsal funiculus(a), CGRP positive terminals in the dorsal horn (b), ChAT positive neurones in the ventral motor horn (c) and NeuN positive neurones in the lateral spinal nucleus (d). Normal EAE disease course is shown at day 29 (N = 5), 58 (N = 5) and 105 (N = 6) (black bars) with early tolerisation (red bar, N = 5) and late tolerisation (green bar, N = 6) being compared to the timepoint when tolerisation occurred and any nerve loss highlighted is significant (P < 0.01, one-way ANOVA). Click here for file

## References

[B1] Compston A Coles A Multiple sclerosis Lancet 2008 1 1502 1517 10.1016/S0140-6736(08)61620-7 18970977

[B2] Rovaris M Confavreux C Furlan R Kappos L Comi G Filippi M Secondary progressive multiple sclerosis: current knowledge and future challenges Lancet Neurol 2006 1 343 354 10.1016/S1474-4422(06)70410-0 16545751

[B3] Confavreux C Vukusic S Age at disability milestones in multiple sclerosis Brain 2006 1 595 605 10.1093/brain/awh714 16415309

[B4] Confavreux C Vukusic S Natural history of multiple sclerosis: a unifying concept Brain 2006 1 606 616 10.1093/brain/awl007 16415308

[B5] Bjartmar C Trapp BD Axonal and neuronal degeneration in multiple sclerosis: mechanisms and functional consequences Curr Opin Neurol 2001 1 271 278 10.1097/00019052-200106000-00003 11371748

[B6] Bjartmar C Trapp BD Axonal degeneration and progressive neurologic disability in multiple sclerosis Neurotox Res 2003 1 157 164 10.1007/BF03033380 12832230

[B7] Dutta R Trapp BD Pathogenesis of axonal and neuronal damage in multiple sclerosis Neurology 2007 1 S22 S31 10.1212/01.wnl.0000275229.13012.32 17548565

[B8] Foote AK Blakemore WF Inflammation stimulates remyelination in areas of chronic demyelination Brain 2005 1 528 539 10.1093/brain/awh417 15699059

[B9] Ruckh JM Zhao JW Shadrach JL van Wijngaarden P Rao TN Wagers AJ Rejuvenation of regeneration in the aging central nervous system Cell Stem Cell 2012 1 96 103 10.1016/j.stem.2011.11.019 22226359 PMC3714794

[B10] Setzu A Lathia JD Zhao C Wells K Rao MS Ffrench-Constant C Inflammation stimulates myelination by transplanted oligodendrocyte precursor cells Glia 2006 1 297 303 10.1002/glia.20371 16856149

[B11] Anderson JM Hampton DW Patani R Pryce G Crowther RA Reynolds R Abnormally phosphorylated tau is associated with neuronal and axonal loss in experimental autoimmune encephalomyelitis and multiple sclerosis Brain 2008 1 1736 1748 10.1093/brain/awn119 18567922

[B12] Baker D O'Neill JK Gschmeissner SE Wilcox CE Butter C Turk JL Induction of chronic relapsing experimental allergic encephalomyelitis in Biozzi mice J Neuroimmunol 1990 1 261 270 10.1016/0165-5728(90)90019-J 2373763

[B13] Hampton DW Anderson J Pryce G Irvine KA Giovannoni G Fawcett JW An experimental model of secondary progressive multiple sclerosis that shows regional variation in gliosis, remyelination, axonal and neuronal loss J Neuroimmunol 2008 1 200 211 18672298 10.1016/j.jneuroim.2008.05.034

[B14] Ludwin SK Sternberger NH An immunohistochemical study of myelin proteins during remyelination in the central nervous system Acta Neuropathol 1984 1 3 240 248 10.1007/BF00685250 6205535

[B15] Manrique-Hoyos N Jürgens T Grønborg M Kreutzfeldt M Schedensack M Kuhlmann T Schrick C Brück W Urlaub H Simons M Merkler D Late motor decline after accomplished remyelination: impact for progressive multiple sclerosis Ann Neurol 2012 1 227 244 10.1002/ana.22681 22367995

[B16] Petzold A Baker D Pryce G Keir G Thompson EJ Giovannoni G Quantification of neurodegeneration by measurement of brain-specific proteins J Neuroimmunol 2003 1 45 48 10.1016/S0165-5728(03)00092-4 12742652

[B17] Pryce G O'Neill JK Croxford JL Amor S Hankey DJ East E Autoimmune tolerance eliminates relapses but fails to halt progression in a model of multiple sclerosis J Neuroimmunol 2005 1 41 52 10.1016/j.jneuroim.2005.04.009 15939483

[B18] Smith PA Morris-Downes M Heijmans N Pryce G Arter E O'Neill JK Epitope spread is not critical for the relapse and progression of MOG 8–21 induced EAE in Biozzi ABH mice J Neuroimmunol 2005 1 76 84 10.1016/j.jneuroim.2005.04.006 15927270

[B19] Al-Izki S Pryce G Jackson SJ Giovannoni G Baker D Immunosuppression with FTY720 is insufficient to prevent secondary progressive neurodegeneration in experimental autoimmune encephalomyelitis Mult Scler 2011 1 939 948 10.1177/1352458511400476 21459808

[B20] Al-Izki S Pryce G O'Neill JK Butter C Giovannoni G Amor S Baker D Practical guide to the induction of relapsing progressive experimental autoimmune encephalomyelitis in the Biozzi ABH mouse Mult Scler Rel Dis 2012 1 29 38 10.1016/j.msard.2011.09.001 25876448

[B21] Hampton DW Seitz A Chen P Heber-Katz E Fawcett JW Altered CNS response to injury in the MRL/MpJ mouse Neuroscience 2004 1 821 832 10.1016/j.neuroscience.2004.05.057 15312895

[B22] Hampton DW Steeves JD Fawcett JW Ramer MS Spinally upregulated noggin suppresses axonal and dendritic plasticity following dorsal rhizotomy Exp Neuro 2007 1 366 379 10.1016/j.expneurol.2006.11.017 17258709

[B23] Scott AL Borisoff JF Ramer MS Deafferentation and neurotrophin-mediated intraspinal sprouting: a central role for the p75 neurotrophin receptor Eur J Neurosci 2005 1 81 92 10.1111/j.1460-9568.2004.03838.x 15654845

[B24] Willis WD Coggeshall RE Sensory Mechanisms of the Spinal Cord 2004 New York: Kluwer Academic/Plenum Publishers

[B25] Jackson SJ Lee J Nikodemova M Fabry Z Duncan ID Quantification of myelin and axon pathology during relapsing progressive experimental autoimmune encephalomyelitis in the Biozzi ABH mouse J Neuropathol Exp Neurol 2009 1 616 625 10.1097/NEN.0b013e3181a41d23 19458548

[B26] Hazama GI Yasuhara O Morita H Aimi Y Tooyama I Kimura H Mouse brain IgG-like immunoreactivity: strain-specific occurrence in microglia and biochemical identification of IgG J Comp Neurol 2005 1 234 249 10.1002/cne.20710 16196032

[B27] Szalai AJ Barnum SR Fc receptors and the common gamma-chain in experimental autoimmune encephalomyelitis J Neurosci Res 2004 1 597 602 10.1002/jnr.20023 14991835

[B28] Readnower RD Chavko M Adeeb S Conroy MD Pauly JR McCarron RM Increase in blood–brain barrier permeability, oxidative stress, and activated microglia in a rat model of blast-induced traumatic brain injury J Neurosci Res 2010 1 3530 3539 10.1002/jnr.22510 20882564 PMC2965798

[B29] Ryu JK McLarnon JG Minocycline or iNOS inhibition block 3-nitrotyrosine increases and blood–brain barrier leakiness in amyloid beta-peptide-injected rat hippocampus Exp Neurol 2006 1 552 557 10.1016/j.expneurol.2005.12.016 16480717

[B30] Schirmer L Albert M Buss A Schulz-Schaeffer WJ Antel JP Brück W Substantial early, but nonprogressive neuronal loss in multiple sclerosis (MS) spinal cord Ann Neurol 2009 1 5 698 704 10.1002/ana.21799 19938172

[B31] Vogt J Paul F Aktas O Müller-Wielsch K Dörr J Dörr S Lower motor neuron loss in multiple sclerosis and experimental autoimmune encephalomyelitis Ann Neurol 2009 1 3 310 322 10.1002/ana.21719 19798635

[B32] Coles A Deans J Compston A Campath-1H treatment of multiple sclerosis: lessons from the bedside for the bench Clin Neurol Neurosurg 2004 1 270 274 10.1016/j.clineuro.2004.02.013 15177782

[B33] Coles AJ Compston DA Selmaj KW Lake SL Moran S Margolin DH Alemtuzumab vs. interferon beta-1a in early multiple sclerosis N Engl J Med 2008 1 1786 1801 18946064 10.1056/NEJMoa0802670

[B34] Bruce CC Zhao C Franklin RJ Remyelination - An effective means of neuroprotection Horm Behav 2010 1 1 56 62 10.1016/j.yhbeh.2009.06.004 19538961

[B35] Franklin RJ Ffrench-Constant C Edgar JM Smith KJ Neuroprotection and repair in multiple sclerosis Nat Rev Neurol 2012 1 11 624 634 10.1038/nrneurol.2012.200 23026979

[B36] Irvine KA Blakemore WF Remyelination protects axons from demyelination- associated axon degeneration Brain 2008 1 1464 1477 10.1093/brain/awn080 18490361

[B37] Hampton DW Innes N Merkler D Zhao C Franklin RJ Chandran S Focal immune-mediated white matter demyelination reveals an age-associated increase in axonal vulnerability and decreased remyelination efficiency Am J Pathol 2012 1 1897 1905 10.1016/j.ajpath.2012.01.018 22426338

[B38] Shen S Sandoval J Swiss VA Li J Dupree J Franklin RJ Casaccia-Bonnefil P Age-dependent epigenetic control of differentiation inhibitors is critical for remyelination efficiency Nat Neurosci 2008 1 9 1024 1034 10.1038/nn.2172 19160500 PMC2656679

[B39] Shields S Gilson J Blakemore W Franklin R Remyelination occurs as extensively but more slowly in old rats compared to young rats following fliotoxin-induced CNS demyelination Glia 1999 1 1 77 83 10.1002/(SICI)1098-1136(199910)28:1<77::AID-GLIA9>3.0.CO;2-F 10594929

