# Supplementary figures and images for: Neurodegeneration progresses despite complete elimination of clinical relapses in a mouse model of multiple sclerosis
Source: Acta Neuropathol Commun. 2013 Dec 23;1:84. doi: 10.1186/2051-5960-1-84 (PMC3895761; doi:10.1186/2051-5960-1-84)

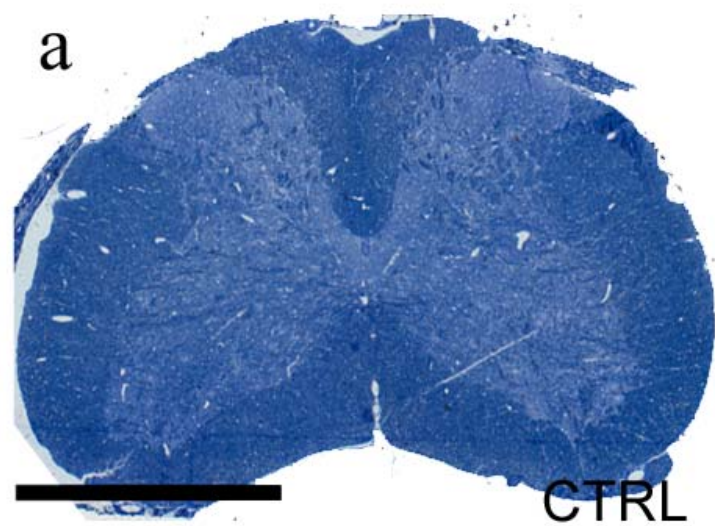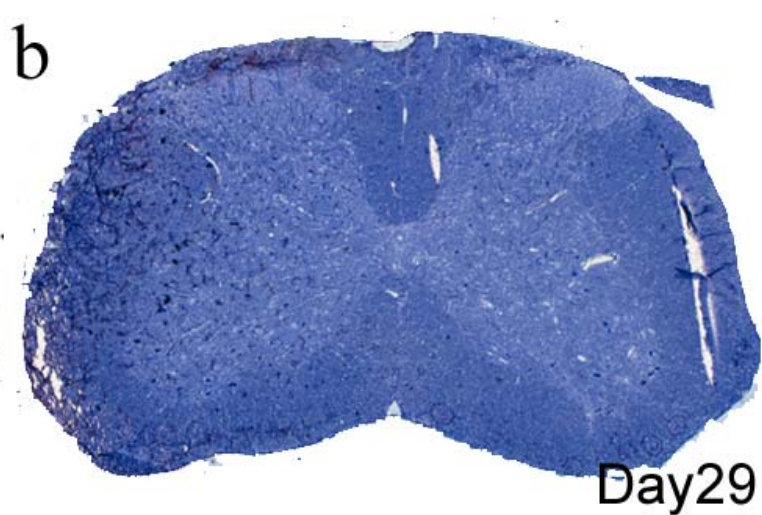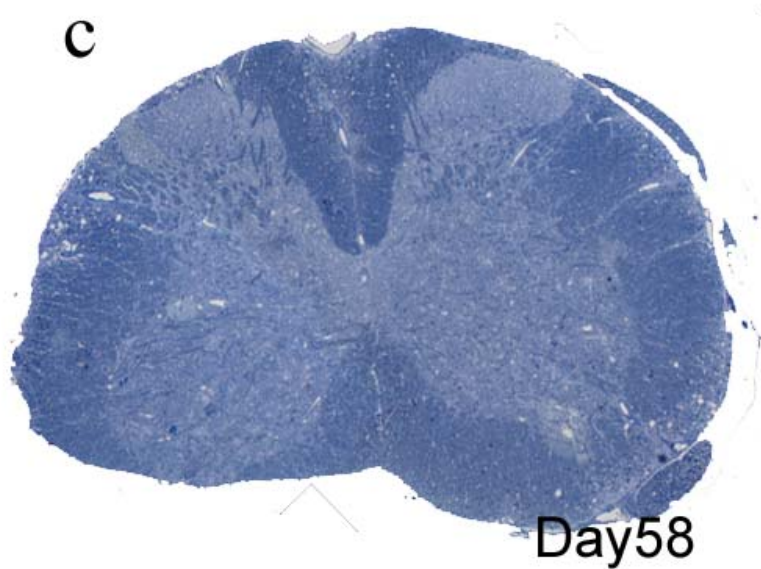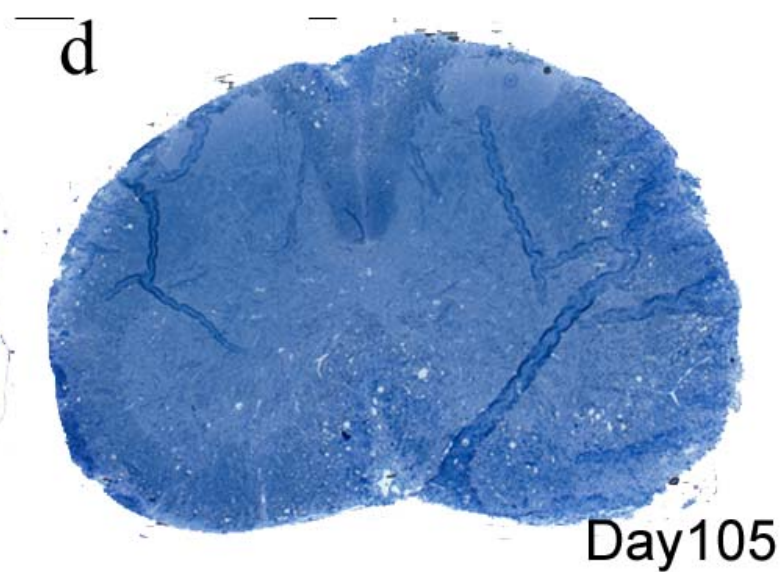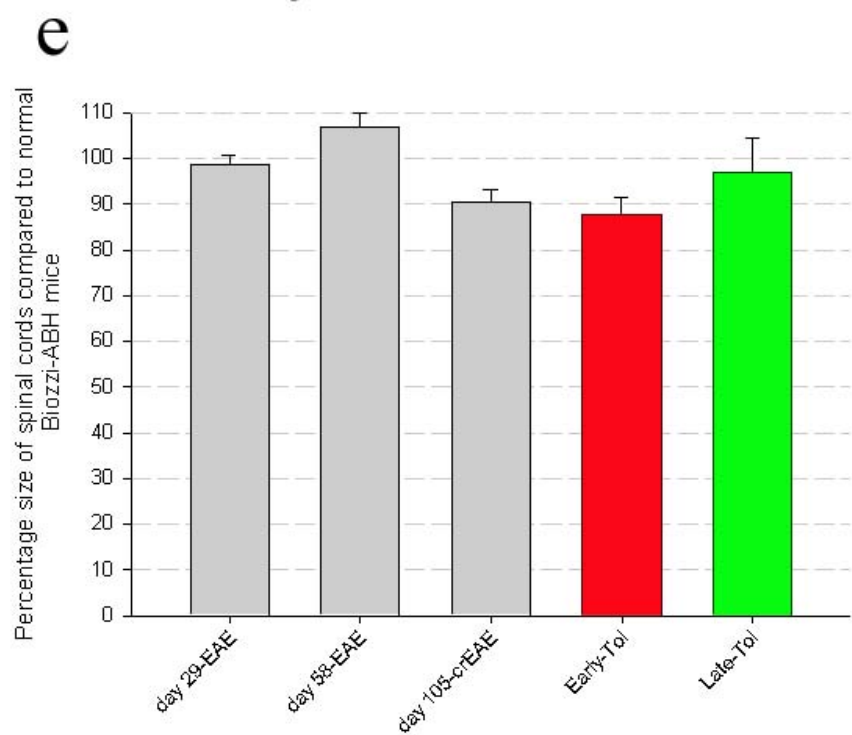

Supplement: Additional file 1: Figure S1 — Representative semithin image sections and graph showing that total area measurements of the spinal cord at all time points are not significantly different therefore tissue oedema and/or atrophy cannot account for the observed changes in axonal counts. Representative semithin images from normal (a, N = 3), day 29 (b, N = 4), day 58 (c; N= ) and day 105-crEAE (d, N = 3). Quantification (e) shows no significant differences between any timepoint nor following early or late tolerisation. Scale bar = 1000 μm for all images. [file 2051-5960-1-84-S1.pdf]

a

## dorsal funiculus

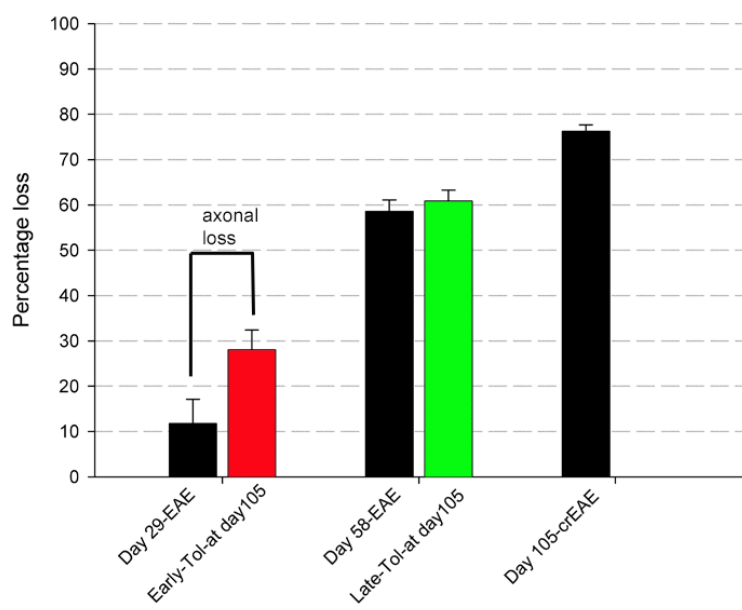

b

## dorsal horn

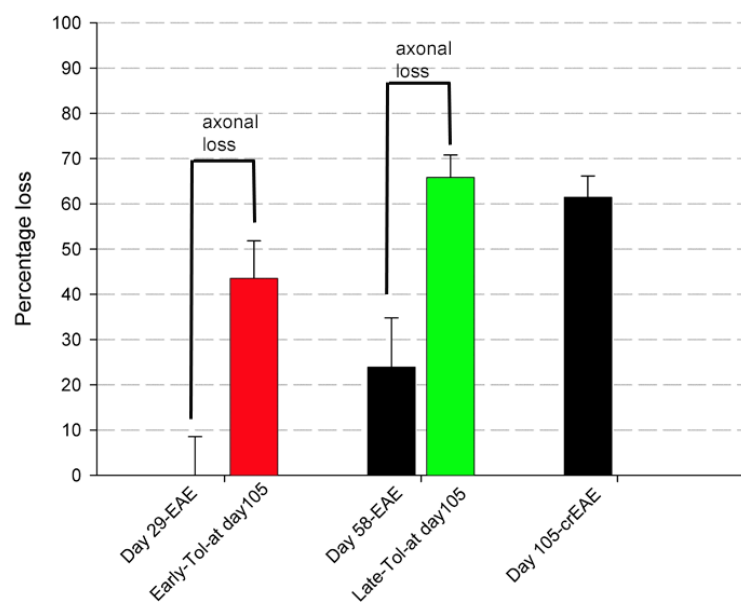

c

## ventral horn

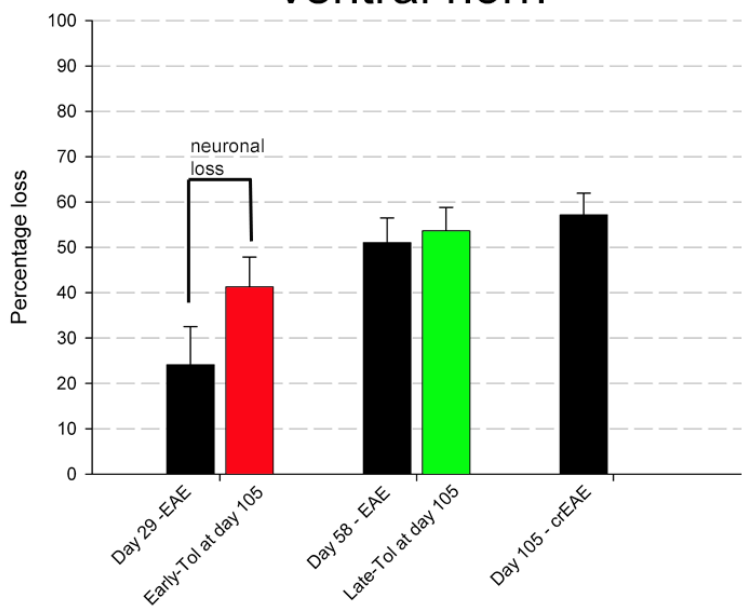

d

## Lateral spinal nucleus

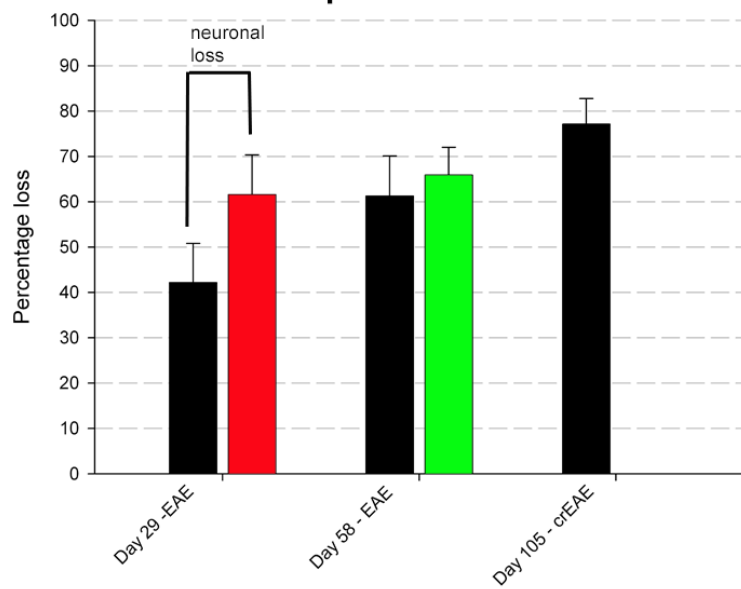

Supplement: Additional file 2: Figure S2 — Graphs showing differences between percentage loss of axons and neurones as disease progresses and following tolerisation. Graphs showing varied axonal (a and b) and neuronal (c and d) loss using quantified semithin axonal counts in the dorsal funiculus(a), CGRP positive terminals in the dorsal horn (b), ChAT positive neurones in the ventral motor horn (c) and NeuN positive neurones in the lateral spinal nucleus (d). Normal EAE disease course is shown at day 29 (N = 5), 58 (N = 5) and 105 (N = 6) (black bars) with early tolerisation (red bar, N = 5) and late tolerisation (green bar, N = 6) being compared to the timepoint when tolerisation occurred and any nerve loss highlighted is significant (P < 0.01, one-way ANOVA). [file 2051-5960-1-84-S2.pdf]
